# Breast and Cervical Cancer Gaps in Displaced Lebanese Women in Syria

**DOI:** 10.1001/jamanetworkopen.2025.25652

**Published:** 2025-08-06

**Authors:** Ahmad Al-Bitar, Ayla Kouli, Omran Janoud, Ali Harb, Hassan Fawaz, Maher Saifo

**Affiliations:** 1 Faculty of Medicine, Damascus University, Damascus, Syrian Arab Republic; 2 Department of Oncology, Albairouni University Hospital, Faculty of Medicine, Damascus University, Damascus, Syrian Arab Republic

## Abstract

**Importance:**

Breast and cervical cancers are leading causes of cancer mortality in low-resource countries, yet awareness remains critically understudied among displaced populations in humanitarian crises. Lebanese women displaced to Syria represent a group navigating compounded vulnerabilities associated with conflict, displacement, and a collapsed health care system and may experience important gaps in cancer knowledge and access.

**Objective:**

To evaluate knowledge of breast and cervical cancer risk factors and symptoms and screening for early cancer detection among displaced Lebanese women in Syria.

**Design, Setting, and Participants:**

This cross-sectional study measured breast cancer, cervical cancer, and human papillomavirus (HPV) knowledge using a survey containing validated, Arabic-translated scales administered via structured interviews from November to December 2024. Lebanese women (aged ≥18 years) displaced to Syria and visiting outpatient health care facilities in Damascus were included. Women who were not Lebanese and with acute or severe physical or mental health conditions were excluded.

**Exposure:**

Breast cancer and cervical cancer knowledge.

**Main Outcomes and Measures:**

The primary outcome was knowledge of cancer symptoms, risk factors, and screening practices. Analyses included Fisher exact test, Kruskal-Wallis test, Mann-Whitney *U* test, and Spearman rank correlation.

**Results:**

Among 378 displaced Lebanese women in Syria (median [IQR] age, 30 [23-39] years), 196 (51.9%) were married, 187 (49.5%) held a university degree, and 222 (58.7%) were unemployed. Only 85 participants (22.5%) had ever undergone breast imaging, 64 (16.9%) had undergone a Papanicolaou test, 274 (72.5%) rarely or never examined their breasts, and 135 (35.7%) ignored observed breast changes. Knowledge gaps were prominent, with 348 (92.1%) having a low awareness of HPV and only 4 (1.1%) correctly identifying age as an important risk factor for breast cancer. Higher educational attainment was significantly associated with improved screening rates and knowledge of breast and cervical cancer (χ^2^_3_ = 11.661; *P* = .009), whereas financial status showed no association.

**Conclusions and Relevance:**

These findings suggest that displaced women during humanitarian crises may face substantial breast and cervical cancer screening barriers, underscoring the need for crisis-responsive health care.

## Key Points

**Question:**

What are the barriers to breast and cervical cancer screening among displaced Lebanese women in Syria?

**Findings:**

In this cross-sectional study of 378 women, the majority of participants lacked awareness of breast and cervical cancer symptoms and signs, with low numbers ever having undergone screening. Higher educational attainment was associated with improved cancer knowledge and screening rates.

**Meaning:**

These findings suggest that targeted interventions are urgently needed to overcome cancer screening barriers in humanitarian crisis settings.

## Teaser

This cross-sectional study evaluates knowledge of breast and cervical cancer risk factors and symptoms and screening for early cancer detection among displaced Lebanese women in Syria.

## Introduction

Breast cancer remains the most diagnosed cancer and a leading cause of cancer mortality globally.^1^ According to GLOBOCAN, in 2020, more than one-half of breast cancer diagnoses and two-thirds of breast cancer–related deaths occurred in low-resource countries.^2^ Several factors contribute to the international variation of the incidence of breast cancer, including early detection; prevalence of established risk factors, including overweight and obesity; use of menopausal hormone therapy; physical inactivity; and alcohol consumption.^3^ Early detection and screening programs should aim to reduce the number of women diagnosed with breast cancer at late stages to less than 30%, according to the World Health Organization.^4^

Furthermore, in countries with fewer resources, cervical cancer ranks as the second most diagnosed cancer and the third leading cause of cancer-related death in female individuals. Approximately 90% of deaths from cervical cancer have occurred in low-resource countries.^2^ Lack of awareness of cervical cancer among the population and limited access to high-quality health care services and cervical screening programs lead to late diagnosis and poor prognosis.^5^ Raising awareness of cervical cancer symptoms and signs might increase people’s ability to detect them early.^6^

Studies on breast and cervical cancer are minimally conducted during humanitarian emergencies.^7^ Over the years, the refugee crisis has affected low-income and low-resource countries.^8^ For the past decade, countries in the Middle East, including Lebanon and Syria, have experienced a long-lasting refugee situation.^9^ In populations from low-resource countries, there is a lack of awareness, diagnostic techniques, and early detection methods of breast and cervical cancer. Concurrently, Syria’s diagnostic capacity, particularly imaging and histopathology, requires reinforcement to enable early detection. Previous research primarily focused on the health status of Syrian and non-Syrian refugees living abroad in various host countries. However, in this study, the first in our knowledge, has taken a different approach by examining awareness and knowledge of breast and cervical cancer among displaced Lebanese women in Syria, in which the health care system is severely damaged and the economy and health care services might be even more fragile than in Lebanon.^10^

## Methods

### Study Design and Participants

This cross-sectional study evaluated awareness of breast and cervical cancer, the leading causes of cancer mortality in low-resource settings,^11^ among displaced Lebanese women in Damascus, Syria, between November 1 and December 7, 2024. This population faces compounded vulnerabilities, including disrupted health care access and heightened cancer risks after migration.^12,13^ This study was approved by the Biomedical Research Ethics Committees at Damascus University. Written informed consent was obtained from each participant prior to the interviews. This study followed the Strengthening the Reporting of Observational Studies in Epidemiology (STROBE) reporting guideline for cross-sectional studies.

Eligible participants (women aged ≥18 years) were systematically recruited from outpatient departments of a tertiary health care facility, a strategy justified by (1) the impracticality of community sampling in humanitarian crises and (2) the need to prioritize feasibility without compromising ethical rigor.^14^ This facility serves as the main referral and access point for displaced populations, including Lebanese refugees, making it a representative entry portal for this group in Syria. Only participants of Lebanese nationality were included; all other nationalities were excluded. Other exclusion criteria (ie, acute or severe physical or mental health conditions) were applied to safeguard data quality and participant welfare, consistent with protocols for research involving displaced populations.^15^

All interviews were conducted face to face in a private room within the facility to ensure confidentiality and comfort. Participation was voluntary.

### Data Collection Tool

We used a survey containing validated scales to measure participants’ knowledge levels of breast cancer (Breast Cancer Awareness Measure^16^), cervical cancer (Cervical Cancer Knowledge Scale^17^), and human papillomavirus (HPV) (HPV Testing Knowledge Scale^18^). Details about these scales and the values of the Cronbach α-coefficients measuring internal consistency for each one are discussed in depth in eMethods 1 in Supplement 1. For this study, all scales were translated from English to Arabic using a dual-direction translation protocol to ensure conceptual equivalence and minimize linguistic bias. Bilingual experts first translated the instruments into Arabic, followed by independent back translation into English. Surveys were administered through structured in-person interviews conducted in a private setting to minimize misunderstanding and improve accuracy. Moreover, and to maintain methodological consistency, trained physicians (A.A.-B., A.H., and H.F.) conducted all interviews to ensure consistency and ethical rigor.

### Sample Size Calculation

The sample size was calculated using OpenEpi, version 3.01 software (Emory University) with a 95% CI, 5% margin of error, and an assumed 50% prevalence rate for breast cancer awareness, cervical cancer knowledge, and HPV testing knowledge. The 50% prevalence estimate was chosen as it yields the maximum sample size in prevalence studies when no prior estimate is available, ensuring sufficient statistical power.

This process yielded a minimum required sample size of 378 participants. Prior to full implementation, a pilot study involving 30 participants was conducted to assess feasibility. Feedback confirmed the practicality of the questionnaire design and logistical procedures.

### Statistical Analysis

Categorical variables are summarized as frequencies and percentages, while continuous variables are expressed as medians with interquartile ranges due to nonnormal distribution. Normal distribution was tested through kurtosis and skewness. To ensure accurate measurement of gaps in breast and cervical cancer awareness, answers to multiple-choice questions were summarized using bar charts. Kruskal-Wallis test was used to compare level of awareness between participants after categorizing them by financial status and educational level. Pairwise comparisons were used to compare differences between groups. Mann-Whitney *U* test was used to compare the level of knowledge between 2 groups. Data were analyzed using SPSS, version 25 (IBM Corp). Results were considered statistically significant at *P* < .05.

## Results

Our study included 378 Lebanese women who were displaced to Syria (median [IQR] age, 20 [23-39] years). More than one-half of the participants were married (196 [51.9%]). The financial status of the sample was predominantly classified as mild income (ie, sufficient for basic needs but not luxuries) (169 participants [44.7%]) and moderate (sufficient for basics and some luxuries) (152 participants [40.2%]); only 40 participants (10.6%) reported low income, and 17 (4.5%) reported high income. Among all participants, 224 (59.3%) had relatives working in the medical field, 187 (49.5%) held a university degree, and 222 (58.7%) were unemployed. Conversely, only 59 participants (15.6%) had not received any formal education. Only 85 participants (22.5%) had previously undergone breast imaging (either mammogram or ultrasound), 64 (16.9%) had undergone a Papanicolaou test prior to the study, 274 (72.5%) rarely or never examined their breasts, and 135 (35.7%) ignored observed breast changes. Further details regarding sociodemographic characteristics are presented in Table 1.

**Table 1.** Participant Sociodemographic Characteristics (N = 378)

Characteristic	Participants, No. (%)
Age, median (IQR), y	30 (23-39)
Marital status	
Single	167 (44.2)
Married	196 (51.9)
Divorced	14 (3.7)
Widowed	1 (0.3)
Financial status	
Low income (insufficient even for basics)	40 (10.6)
Mild income (sufficient for basics but not luxuries)	169 (44.7)
Moderate income (sufficient for basics and some luxuries)	152 (40.2)
High income (sufficient for everything)	17 (4.5)
Relative working in the medical field	
No	154 (40.7)
Yes	224 (59.3)
No. of children	
0	194 (51.3)
1 or 2	88 (23.3)
≥3	96 (25.4)
Breastfed children	
No	202 (53.4)
Yes	176 (46.6)
Area of residence	
Countryside	165 (43.7)
City	213 (56.3)
Education	
None or had not completed secondary school	59 (15.6)
Secondary school	104 (27.5)
University degree	187 (49.5)
Master’s degree or higher	28 (7.4)
Employment	
Do not work	222 (58.7)
Work in an office (part time)	31 (8.2)
Work in an office (full time)	44 (11.6)
Work in a field that requires physical power (part time)	44 (11.6)
Work in a field requires physical power (full time)	37 (9.8)
Have had a mammogram or breast ultrasound	
No	293 (77.5)
Yes	85 (22.5)
Have had a Papanicolaou test	
No	314 (83.1)
Yes	64 (16.9)

The χ^2^ test indicated no significant association between the financial status of participants and their prior receipt of either breast imaging or Papanicolaou tests. However, there was a significant association between the educational level of participants and having had a Papanicolaou test (χ^2^_3_ = 11.661; *P* = .009), with those having a higher education level showing a greater likelihood of having undergone a Papanicolaou test previously. Similarly, participants with higher educational levels exhibited a higher proportion of having undergone mammograms or breast ultrasounds compared with those with lower educational attainment (χ^2^_3_ = 24.051; *P* < .001).

When inquiring whether participants or their partners had been diagnosed with cancer, we found that the majority did not report such diagnoses. However, 253 participants (66.9%) indicated that at least 1 relative had a history of cancer, and 101 (26.7%) reported having at least 1 friend with a history of cancer (eFigure 1 in Supplement 1).

With the Breast Cancer Awareness Measure, we identified a significant gap in knowledge among participants regarding the warning signs of breast cancer. Only a small number of participants were unaware that symptoms of breast cancer included thickening in the breast (68 [18.0%]), thickening or lump under the armpit (75 [19.8%]), bleeding or discharge from the nipple (77 [20.4%]), and changes in breast shape (193 [51.1%]) (eFigure 2 in Supplement 1); only 33 (8.7%) reported checking at least once per month. A total of 213 participants (56.3%) expressed moderate confidence in their ability to notice changes when they occurred; however, among those who had previously noticed changes in their breasts, 135 (35.7% of the total sample) did not consult a physician after such observations (Table 2). Despite this trend, when asked about future intentions regarding seeking medical assistance upon noticing changes in their breasts, 138 participants (36.5%) stated that they would seek help immediately if such changes occurred again; conversely, 25 participants (6.6%) indicated that they would refuse to consult a physician. To assess knowledge concerning increased risk factors for breast cancer associated with advancing age, only 4 participants (1.1%) responded correctly to the question, “In the next year, who is most likely to develop breast cancer?”

**Table 2.** Attitudes Toward Seeking Physician Help and Examining the Breast (N = 378)

Question	Participants, No. (%)
How often do you check your breasts?	
At least once a week	15 (4.0)
At least once a month	33 (8.7)
At least once every 6 mo	56 (14.8)
Rarely or never	274 (72.5)
Are you confident you would notice a change in your breasts?	
Very confident	19 (5.0)
Fairly confident	213 (56.3)
Not very confident	103 (27.2)
Not at all confident	43 (11.4)
Have you ever been to see a doctor about a change you have noticed in one of your breasts?	
Yes	79 (20.9)
No	135 (35.7)
Never noticed a change in one of my breasts	164 (43.4)
If you found a change in your breast, how soon would you contact your doctor?	
Immediately	138 (36.5)
During the first 48 h	98 (25.9)
After 2 d but within the following week	67 (17.7)
After 1 wk but within the following 2 wk	17 (4.5)
After 2 wk but within the following month	23 (6.1)
After more than a month	7 (1.9)
Until observing changes in the menstrual cycle or at the end of the menstrual cycle	3 (0.8)
Will not consult a doctor	25 (6.6)
In the next year, who is most likely to develop breast cancer?	
A woman of any age^a^	175 (46.3)
A woman aged 70 y^b^	4 (1.1)
A woman aged 50 y^a^	119 (31.5)
A woman aged 30 y^a^	80 (21.2)

^a^ Incorrect answer.

^b^ Correct answer.

To investigate the association between participants’ confidence levels and their accuracy in responding to the question, “In the next year, who is most likely to develop breast cancer,” the Fisher exact test was used. The analysis revealed no significant association between these 2 variables. This finding suggests that the degree of confidence expressed by participants may not be directly linked to their awareness of age as a risk factor for breast cancer. Additionally, Fisher exact test was used to examine the association between participants’ confidence and their likelihood of seeking medical assistance upon noticing changes in their breasts. The results indicated no significant association, underscoring that confidence in recognizing changes does not necessarily correlate with the propensity to seek help.

**Figure 1.**
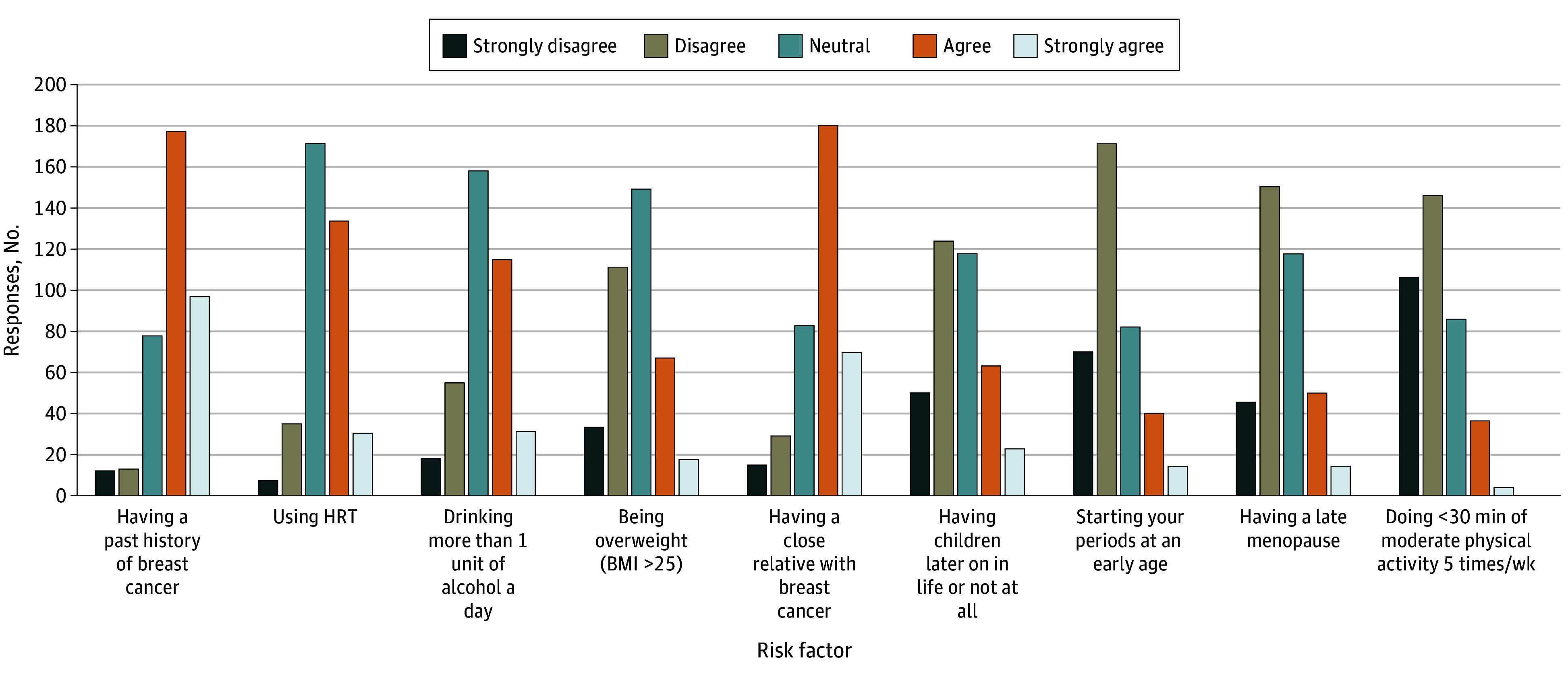
Distribution of Answers Regarding the Item Testing Knowledge About Predisposing Risk Factors BMI indicates body mass index (calculated as weight in kilograms divided by height in meters squared); HRT, hormone replacement therapy.

Furthermore, an assessment of participants’ agreement regarding well-established risk factors for breast cancer revealed a notable gap in knowledge about the roles of physical inactivity, late menopause, early menarche, and delayed childbirth in breast cancer development. A substantial proportion of participants maintained a neutral stance when questioned about the contributions of hormone replacement therapy, obesity, and excessive alcohol consumption (defined as >1 unit per day) to breast cancer risk (Figure 1).

Overall, participants showed mild to moderate knowledge regarding cervical cancer, with 218 (57.7%) answering at least half of the questions incorrectly. On the other hand, while 340 (90.0%) answered at least half of the question investigating HPV understanding incorrectly. The distribution of scores are shown in eFigure 3 in Supplement 1. In examining the Spearman correlation coefficients for knowledge of HPV and cervical cancer relative to participant age, a small negative association was identified (*r* = −0.103; *P* = .045). However, no significant association was found between age and knowledge of cervical cancer. A positive association was observed between knowledge of HPV and cervical cancer overall (*r* = 0.458; *P* < .001) (eTable in Supplement 1).

**Figure 2.**
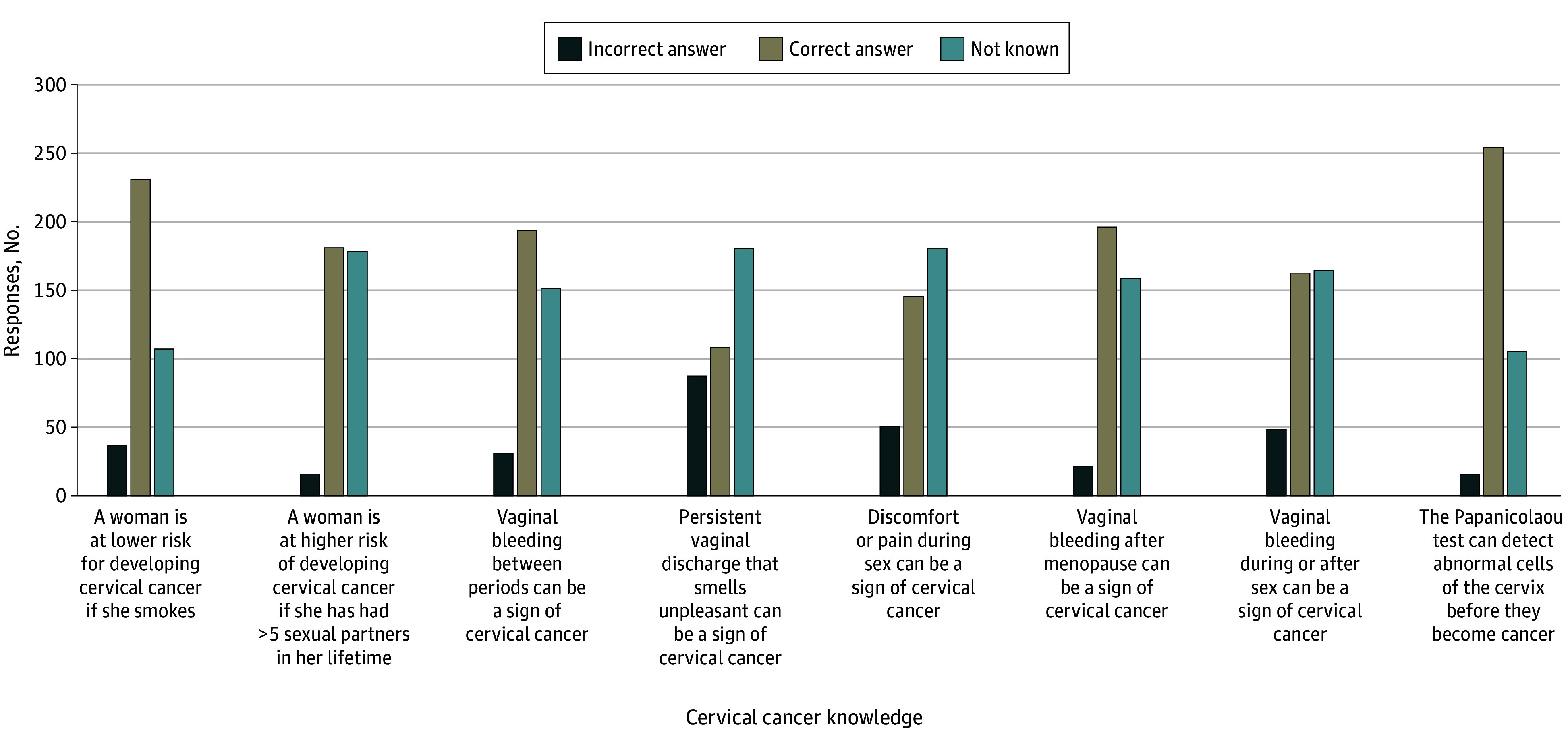
Knowledge of Each Item on the Cervical Cancer Knowledge Scale

Analysis of responses to the Cervical Cancer Knowledge Scale indicated that many participants overlooked key signs associated with cervical cancer, such as unpleasant vaginal discharge and discomfort or pain during intercourse. However, most participants answered other questions correctly (Figure 2).

**Figure 3.**
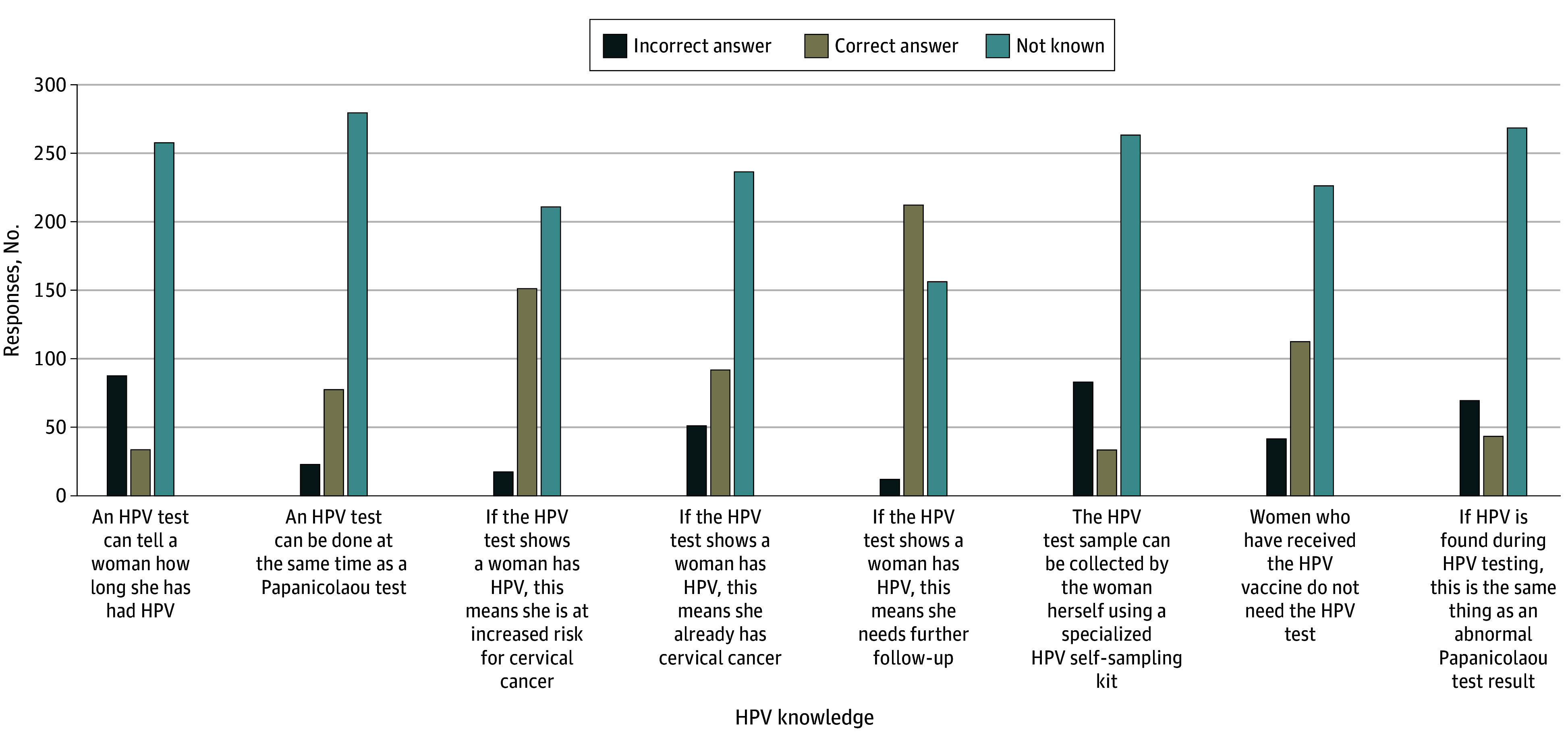
Knowledge of Each Item on the Human Papillomavirus (HPV) Testing Knowledge Scale

The majority of participants exhibited limited knowledge regarding HPV, as evidenced by high percentages of incorrect responses across various HPV Testing Knowledge Scale questions. Notably, only 1 question, “If an HPV test shows a woman has HPV, does this mean she needs further follow-up,” was answered correctly by 212 (56.1%) of the participants. For all other questions on this scale, participants showed substantial gaps in understanding (Figure 3).

To investigate how knowledge regarding cervical cancer and HPV differs between different groups within the sample, Kruskal-Wallis test, pairwise comparisons, and Mann-Whitney *U* tests were conducted. A detailed discussion about these findings is provided in eMethods 2 in Supplement 1.

## Discussion

Breast cancer and cervical cancer both remain a worldwide public health problem. Women’s awareness of these types of cancers plays an important role in the recognition and progression of breast and cervical cancer.^19^ This cross-sectional study was conducted during a humanitarian emergency to assess the awareness of Lebanese women in Syria about breast and cervical cancer. According to WorldData.info, both Syria and Lebanon are listed among low-resource countries.^20,21^ A big difference in breast cancer survival rates is seen between high-resource and low-resource countries.^22^ Furthermore, studies on cancer and the control of cancer during humanitarian emergencies are minimal compared with communicable diseases.^23^ The health care system in Syria is heavily affected by the decade-long Syrian conflict, and it is challenging for citizens to gain access to treatment and care.^10^ Syria’s health care workforce, facilities, and supply chains have been decimated, depriving 70% of the population of reliable care. For patients with cancer, this collapse is lethal. Mammography, Papanicolaou tests, and palliative care are virtually nonexistent, and public health campaigns on cancer risks and symptoms have dissolved.

In Arab countries, breast cancer occurs at an earlier age compared with Western countries.^24^ The median age of our studied sample was 30 years. There is a higher incidence of breast cancer in Lebanon compared with other Arab countries.^25^ Our sample included Lebanese women who were recently displaced to Syria. Participants showed a substantial knowledge gap regarding breast cancer warning signs, unlike greater awareness in other low-resource countries.^26^ Substantial knowledge gaps existed regarding breast cancer risk factors (physical inactivity, late menopause, early menarche, and delayed childbirth). This low awareness underscores the need for health education tailored to population literacy levels. Early detection requires public awareness programs, but barriers include lack of education and cultural inhibitions around breast health in Arab countries such as Syria and Lebanon.

On the other hand, by 2030, cervical cancer may cause the death of approximately 500 000 women annually, with more than 95% of these deaths occurring in low- and middle-income countries.^27^ However, there is evidence that cervical cancer screening contributes to the reduction in the development of cervical cancer and the associated mortality rate.^28^ In low-resource countries, screening programs for cervical cancer face some obstacles, including a lack of awareness of the benefits of cervical screening due to poor public health education, sociocultural health beliefs, and gender roles.^29^ There is insufficient research on knowledge access to cervical cancer screening among Arab women in the Middle East.^30^ The uptake of screening methods in Arab women is associated with the knowledge of the clinical features of cervical cancer.^31^ The Papanicolaou test is the main cervical cancer screening tool and is used to detect precancerous stages of endocervical cancer.^32^ On the Cervical Cancer Knowledge Scale used to evaluate the knowledge of our sample about cervical cancer, most participants knew that a Papanicolaou test is an early detection method. However, there was no statistically significant difference between those who had undergone a Papanicolaou test and those who had not in terms of knowledge of cervical cancer. A lack of knowledge of cervical cancer screening has been observed in neighboring countries, including Iraq, Jordan, and Kuwait.^33^ According to the National Cancer Institute, early symptoms of cervical cancer include vaginal bleeding after sex, after menopause, or between periods; vaginal discharge that is watery and has a strong odor or that contains blood; and pelvic pain or pain during sex. The majority of our participants were not aware that persistent vaginal discharge and pain during sex could be signs of cervical cancer.

In Arab countries, the prevalence of HPV is 16% in the general population and 80% in patients with cervical cancer.^34^ Persistent high-risk HPV infection is the main cause of cervical cancer, and HPV subtypes 16 and 18 are associated with more than 70% of cervical cancer cases.^35^ We aimed to evaluate the knowledge of HPV in our participants due to its association with cervical cancer. On the HPV Testing Knowledge Scale, the majority of participants answered not known for 7 of the 8 questions. When studying the correlation between the knowledge of HPV and cervical cancer and the age of the participant, we noticed that there was a small negative association between age and knowledge of HPV, consistent with other studies.^36^ This association may be attributed to HPV tending to affect young sexually active women. The knowledge of HPV differs between participants when categorizing them depending on their financial status. Participants with a high income had more knowledge compared with those with a low or mild income. However, no association was observed between age and knowledge of cervical cancer. There was a positive association between the knowledge of HPV and cervical cancer. However, the knowledge of both was higher in individuals with higher educational attainment, which highlights the importance of education and knowledge in the prevention and early detection of cervical cancer.^8^

Rebuilding Syria’s health care system requires international investment. Equity policies must guarantee women’s cancer care access. Without change, preventable suffering traps neglected populations. Cancer care is urgent and cannot wait for stability. Our use of outpatient department–based recruitment, while potentially limiting generalizability, reflects the pragmatic realities of conducting research in humanitarian settings, in which community access is often restricted due to security, mobility, or stigma.^37^ Notably, this approach enabled inclusion of a critically understudied population, ie, displaced Lebanese women, whose cancer prevention needs are frequently overlooked in regional health policies. Future studies should use community-engaged methods (eg, snowball sampling via local nongovernmental organizations [NGOs]) to capture hard-to-reach subgroups, though such efforts require sustained funding and trust building.^38^ Addressing these disparities requires comprehensive strategies, including the integration of cancer screening services into primary health care, culturally sensitive educational campaigns, and the training of community health workers. Collaborations between governmental organizations and NGOs are essential to ensure the sustainability of these interventions.^39^

### Strengths and Limitations

This study offers important contributions as the first that we know of to assess breast and cervical cancer awareness among displaced Lebanese women in Syria, a population historically overlooked in humanitarian oncology research. Its strengths include the use of validated, culturally adapted tools with high internal consistency and a comprehensive analysis integrating cancer knowledge, screening behaviors, and displacement-related barriers.

The study also has some limitations. The cross-sectional design limits causal interpretations, and reliance on self-reported data risks recall bias. The narrow focus on Lebanese women in Damascus excludes other vulnerable groups (eg, Syrian refugees, men), potentially limiting generalizability.

Despite these limitations, the findings reveal systemic neglect of cancer care in crises and call for urgent interventions such as mobile clinics and NGO screenings. Future research must use robust mixed methods to overcome cultural and structural barriers. The study’s narrow demographics limited generalizability, so subsequent work should prioritize longitudinal designs and broader participation to deepen understanding and enable targeted interventions for displaced populations.

## Conclusions

This cross-sectional study exposes an urgent and preventable crisis in women’s health care among displaced populations, marked by life-threatening deficits in breast and cervical cancer awareness. While Lebanese women have some of the region’s highest breast cancer rates, 98.9% of our participants did not recognize aging as a risk factor and 72.5% never examined their breasts. Furthermore, HPV knowledge, a cornerstone of cervical cancer prevention, was virtually nonexistent. These gaps are not merely in knowledge; they are consequences of systemic neglect and institutionalized inequity. The disparity is stark. While screening rates exceed 80% in high-income countries,^40^ displaced women in humanitarian settings are too often left behind. Policymakers and health systems must prioritize mobile cancer screening, culturally adapted HPV vaccination, and trained female community health workers for early detection. Partnerships with local NGOs and health ministries are essential for sustainable programs. Cancer care must be a public health priority, even in crises. Without prompt action, displaced women could face preventable cancers, an ethically unacceptable outcome.

## Notes

Supplement 1. **eMethods 1.** Details Regarding Data Collection Tool **eFigure 1.** Having a Previous or Current History of Cancer **eFigure 2.** Distribution of the Answers Regarding Each Item Testing Knowledge Toward Warning Signs of Breast Cancer **eFigure 3.** Overview of Overall Knowledge of Cervical Cancer and Human Papillomavirus Infection **eTable.** Summary of Correlation Analysis **eMethods 2.** Details Regarding Comparisons Between Different Categories of Participants Using Kruskal-Wallis Test, Pairwise Comparisons, and Mann-Whitney Test

Supplement 2.
Data Sharing Statement
