# Supplementary material for: Breast and Cervical Cancer Gaps in Displaced Lebanese Women in Syria
Source: JAMA Netw Open. 2025 Aug 6;8(8):e2525652. doi: 10.1001/jamanetworkopen.2025.25652 (PMC12329606; doi:10.1001/jamanetworkopen.2025.25652)
Supplement: Supplement 1. — eMethods 1. Details Regarding Data Collection Tool eFigure 1. Having a Previous or Current History of Cancer eFigure 2. Distribution of the Answers Regarding Each Item Testing Knowledge Toward Warning Signs of Breast Cancer eFigure 3. Overview of Overall Knowledge of Cervical Cancer and Human Papillomavirus Infection eTable. Summary of Correlation Analysis eMethods 2. Details Regarding Comparisons Between Different Categories of Participants Using Kruskal-Wallis Test, Pairwise Comparisons, and Mann-Whitney Test [file jamanetwopen-e2525652-s001.pdf]

Supplemental Online Content

Al-Bitar A, Kouli A, Janoud O, Harb A, Fawaz H, Saifo M. Breast and cervical cancer gaps in displaced Lebanese women amid Syria's health collapse. *JAMA Netw Open*. 2025;8(8):e2525652. doi:10.1001/jamanetworkopen.2025.25652

eMethods 1. Details Regarding Data Collection Tool

eFigure 1. Having a Previous or Current History of Cancer

eFigure 2. Distribution of the Answers Regarding Each Item Testing Knowledge Toward Warning Signs of Breast Cancer

eFigure 3. Overview of Overall Knowledge of Cervical Cancer and Human Papillomavirus Infection

eTable. Summary of Correlation Analysis

eMethods 2. Details Regarding Comparisons Between Different Categories of Participants Using Kruskal-Wallis Test, Pairwise Comparisons, and Mann-Whitney Test

This supplemental material has been provided by the authors to give readers additional information about their work.

© 2025 Al-Bitar A et al. *JAMA Network Open*.

eMethods 1. Details Regarding Data collection Tool

*Breast Cancer Awareness Measure (BCAM):

The Breast Cancer Awareness Measure (BCAM) was developed by the Cancer Research UK group of King's College London in 2009(14). BCAM is a questionnaire that assesses seven domains of BC awareness: knowledge of BC symptoms, breast self-examination, confidence in noticing a change in the breasts, age and lifetime risk of BC, urgency if a change in the breast is noticed, BC risk factors, and BC screening. BCAM is highly readable for patients, has good test-retest reliability and good construct validity, and is sensitive to changes in awareness. BCAM has been validated in Muslim and Middle Eastern populations(15)(16)(17).

Cronbach α coefficient for the section of this scale investigating knowledge of the signs of breast cancer is 0.807 (High internal consistency)

Cronbach α coefficient for the section of this scale investigating knowledge of the risk factors for developing breast cancer is 0.757 (High internal consistency)

*Cervical Cancer Knowledge Scale (CCKS):

The Cervical Cancer Knowledge Scale (CCKS) is a validated instrument designed to assess participants' understanding of cervical cancer, including its etiology, risk factors, prevention strategies (e.g., screening, vaccination), and symptoms. The scale typically consists of 8 items of true/false questions. Example items include knowledge about the role of HPV in cervical cancer, the purpose of Pap smears, and early warning signs of the disease. The CCKS has been widely used in public health research and demonstrates strong psychometric properties. Its validation involved a multi-step process, including content validity assessment by a panel of oncologists and epidemiologists, pilot testing in diverse populations, and internal consistency analysis.(18) Construct validity was established through factor analysis, confirming its ability to distinguish between high and low health literacy groups.

Cronbach α coefficient for the section of this scale is 0.746 (High internal consistency)

*HPV Testing Knowledge Scale (HTKS):

The HPV Testing Knowledge Scale (HTKS) evaluates participants' awareness of HPV testing protocols, interpretation of results, and linkages to cervical cancer prevention. The scale includes 8 items addressing topics such as the recommended age for HPV testing, the significance of co-testing (HPV and Pap smear), and follow-up procedures for positive results. The HTKS was validated in a cross-sectional study by Thompson et al(19), which confirmed its reliability and content validity through expert review. Convergent validity was demonstrated by strong correlations with existing health literacy tools (r = 0.72). The scale has been adapted for use in low-resource settings, with translations and cultural modifications verified via back-translation and cognitive interviews.

Cronbach α coefficient for the section of this scale is 0.864 (High internal consistency)

© 2025 Al-Bitar A et al. *JAMA Network Open*.

eFigure 1. Having a Previous or Current History of Cancer

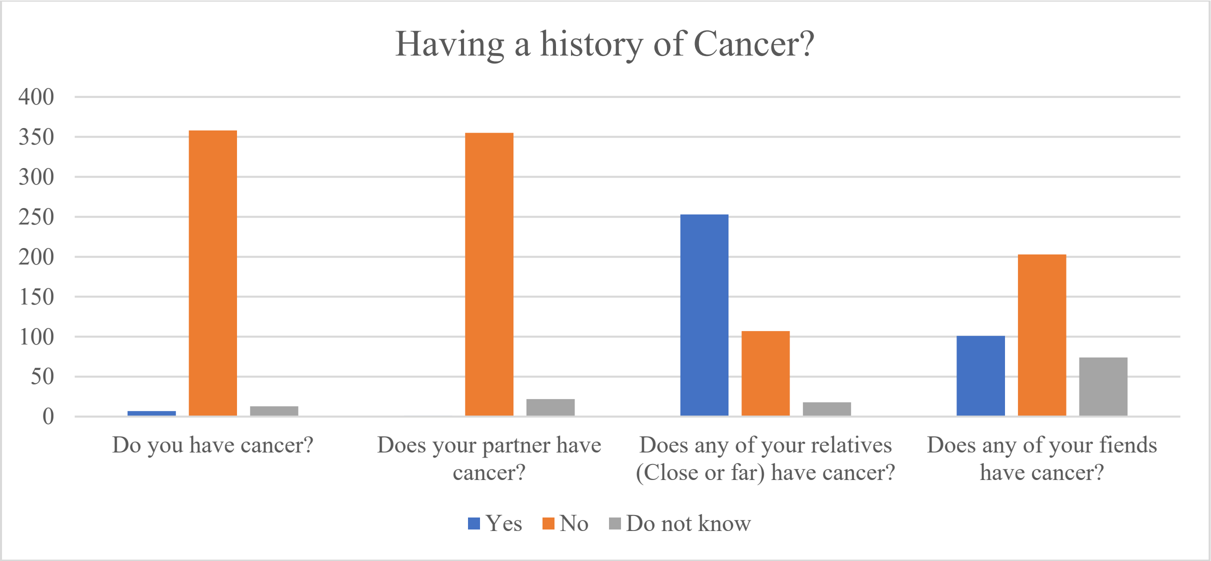

eFigure 2. Distribution of the Answers Regarding Each Item Testing Knowledge Toward Warning Signs of Breast Cancer

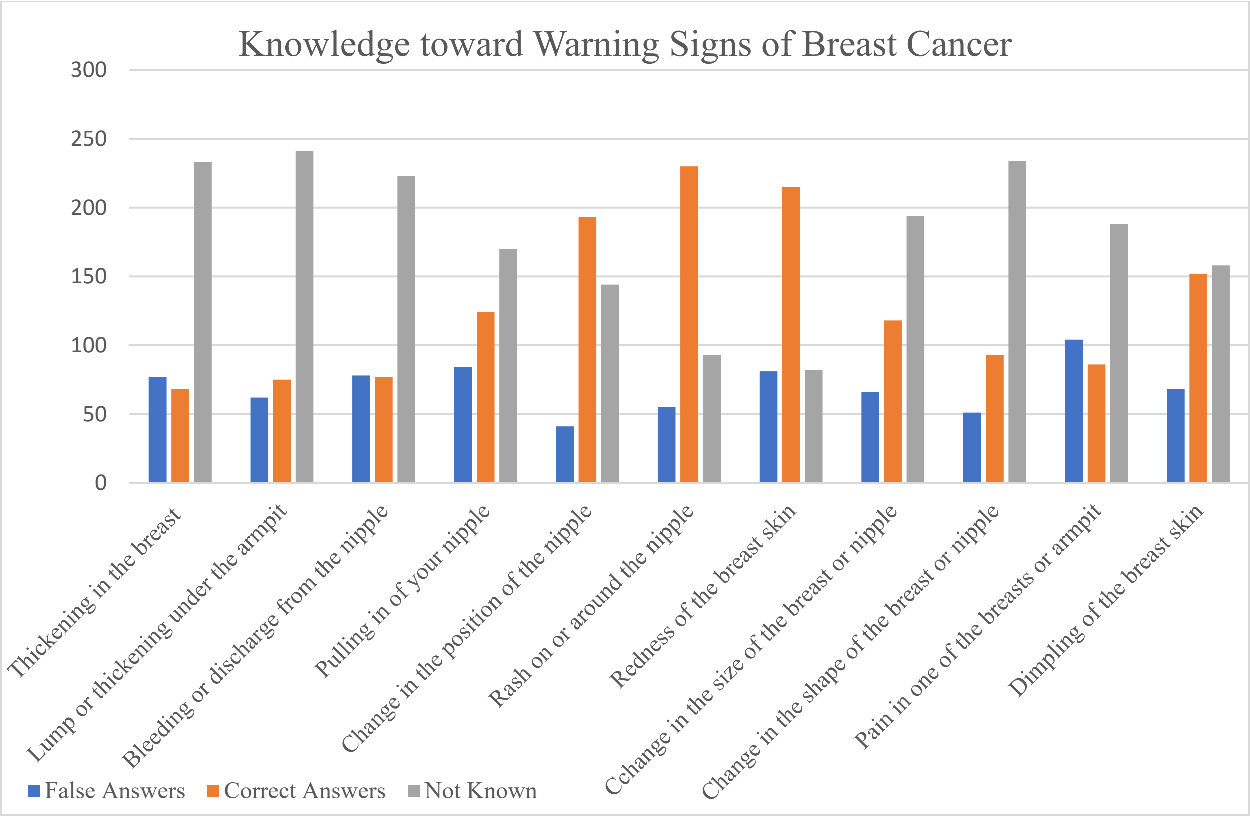

© 2025 Al-Bitar A et al. *JAMA Network Open*.

eFigure 3. Overview of Overall Knowledge of Cervical Cancer and Human Papillomavirus Infection

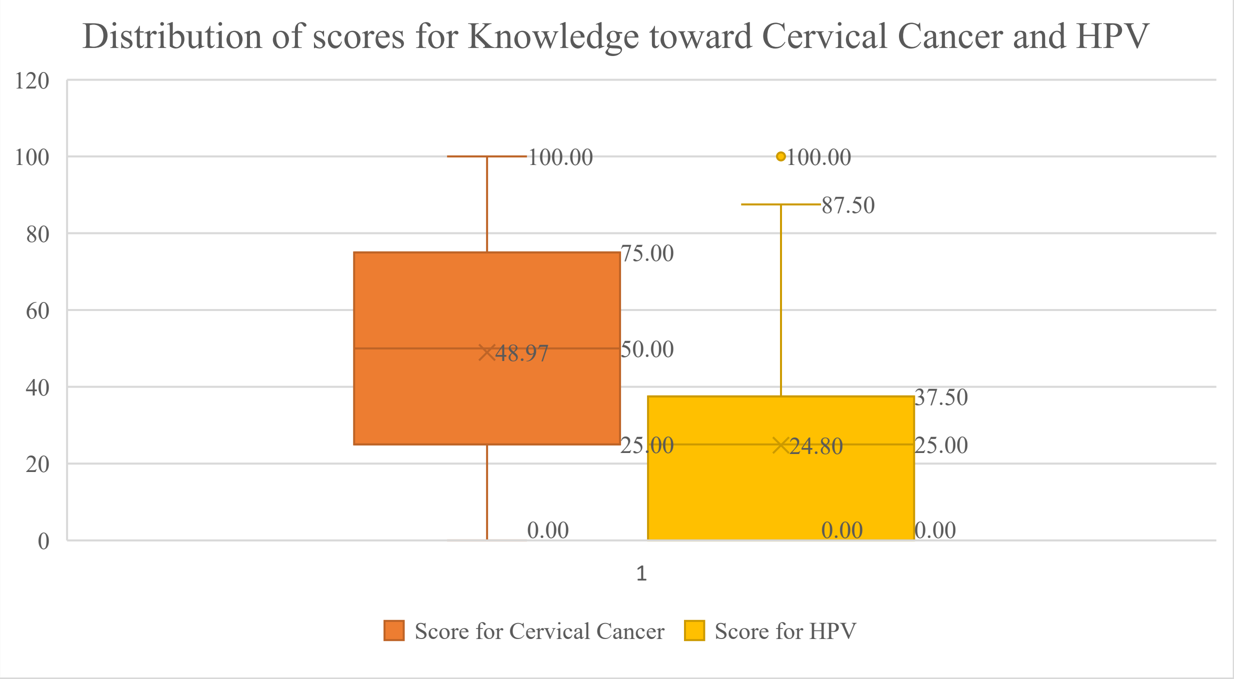

eTable. Summary of the Correlation Analysis (the values reported are Spearman correlation coefficient)

	Knowledge of Cervical Cancer	Knowledge of Human Papillomavirus
Age	- 0.060 (P-Value = 0.245)	**- 0.103 (P-Value = 0.045)**
Knowledge of Cervical Cancer		**+ 0.458 (P-Value = 10^{-19})**

© 2025 Al-Bitar A et al. *JAMA Network Open*.

eMethods 2. Details Regarding Comparisons Between Different Categories of Participants Using Kruskal-Wallis Test, Pairwise Comparisons, and Mann-Whitney Test

Kruskal-Wallis Test indicated that knowledge regarding HPV varies among participants when categorized by financial status (*P-value* < 0.01). Pairwise Comparisons revealed a significant difference in knowledge between individuals with high income and those with low or moderate income (*P-value* < 0.05). However, no significant differences were observed among these categories concerning knowledge about cervical cancer.

In terms of educational background, knowledge regarding both cervical cancer and HPV also differed significantly across categories. The Kruskal-Wallis Test demonstrated a significant difference in knowledge about cervical cancer (*Test Statistic = 7.92, P-value = 0.048*) and HPV (*Test Statistic = 9.93, P-value = 0.019*). Pairwise Comparisons indicated that individuals with higher educational attainment exhibited a better understanding of these topics.

Furthermore, no statistically significant differences were found between residents of rural areas and urban areas regarding their knowledge of both cervical cancer and HPV, as determined by a Mann-Whitney test (*P-value* > 0.05). However, the Mann-Whitney test did reveal a significant difference in HPV knowledge between individuals who have relatives in the medical field and those who do not (*Z = -2.297, P-value = 0.022*). No differences in knowledge regarding cervical cancer were observed based on this criterion.

One of the interesting findings is the lack of statistically significant difference between those who performed a Pap smear before and those who did not in terms of the knowledge of both cervical cancer (*Z = -1.412, P-value = 0.158*) and HPV (*Z = -0.155, P-value = 0.877*).

© 2025 Al-Bitar A et al. *JAMA Network Open*.
