# Supplementary material for: Breast and Cervical Cancer Gaps in Displaced Lebanese Women in Syria
Source: JAMA Netw Open. 2025 Aug 6;8(8):e2525652. doi: 10.1001/jamanetworkopen.2025.25652 (PMC12329606; doi:10.1001/jamanetworkopen.2025.25652)
Supplement: Supplement 2. — Data Sharing Statement [file jamanetwopen-e2525652-s002.pdf]

Data Sharing Statement

Al-Bitar. Breast and Cervical Cancer Gaps in Displaced Lebanese Women Amid Syria's Health Collapse. *JAMA Netw Open*. Published August 06, 2025.
doi:10.1001/jamanetworkopen.2025.25652

Data
Data available: No
